# Application of Traffic Cone Target Detection Algorithm Based on Improved YOLOv5

**DOI:** 10.3390/s24227190

**Published:** 2024-11-09

**Authors:** Mingwu Wang, Dan Qu, Zedong Wu, Ao Li, Nan Wang, Xinming Zhang

**Affiliations:** 1 Department of Mechanical Engineering, Shaanxi University of Technology, Hanzhong 723001, China; wucmsy@163.com (Z.W.); 18211788337@163.com (A.L.); heroyoyu@126.com (N.W.); 2 Hanjiang Thread Grinding Machines Research Institute, Hanjiang Machine Tool Co., Ltd., Hanzhong 723003, China; zhu0916@163.com; 3 Yangxian Guangda New Energy Machinery Co., Ltd., Hanzhong 723300, China; gdzhangxinming@126.com

**Keywords:** road maintenance, target detection, network deployment, deep learning, automatic traffic cone retractor

## Abstract

To improve the automation level of highway maintenance operations, the lightweight YOLOv5-Lite-s neural network was deployed in embedded devices to assist an automatic traffic cone retractor in completing recognition and positioning operations. The system used the lightweight shuffle Net network as a backbone for feature extraction, replaced convolutional layers with focus modules to reduce computational complexity, and reduced the use of the C3 layer to increase network speed, thereby meeting the speed and accuracy requirements of traffic cone placement and retraction operations while maintaining acceptable model inference accuracy. The experimental results show that the network could maintain recognition accuracy and speed values of around 89% and 9 fps under different working conditions such as varying distances, lighting conditions, and occlusions, meeting the technical requirements for deploying and retrieving cones at a speed of 30 cones per minute when the operating vehicle’s speed was 20 km/h. The automatic traffic cone placement and retraction system operated accurately and stably, achieving the application of machine vision in traffic cone retraction operations.

## 1. Introduction

Traffic cones are hollow cone-shaped objects made of plastic or rubber that are used for road closure or emergency construction, as well as lane separation and diversion work [1]. China’s highway mileage ranks first in the world, and traditional road maintenance involves the manual placement and retraction of cones, which is not only time-consuming and labor-intensive, but also a dangerous working environment. In recent years, highway maintenance companies have gradually replaced manual cone placement and retraction with automatic traffic cone retraction, which has not only improved the efficiency of road maintenance work but has also reduced the probability of maintenance workers being involved in accidents.

Currently, most domestic automatic traffic cone retractors are controlled by hydraulic or pneumatic mechanical arms that retract and release cones. These retractors have complex structures, large volumes, and high maintenance costs. In addition, there are few research results on traffic cone machine vision recognition. On the one hand, when an automatic traffic cone retractor is retrieving cones, a cone’s position is generally located by the driver controlling the vehicle’s route, which not only greatly distracts the driver, leading to a decrease in driving safety, but also causes missed cones due to inaccurate cone positioning [2]. On the other hand, the specifications and sizes of traffic cones are different under different working conditions, making universal cone collection inconvenient and often result in cone jamming failures that affect the operation process. Therefore, the industry urgently needs a small integrated automatic traffic cone retractor with simple operation and a target detection system.

Wang L. et al. [3] developed an algorithm that can quickly identify traffic cones based on color and can perceive corresponding distance data. This algorithm can be deployed on embedded devices, but in various scenarios such as extreme weather, nighttime, strong light, curved driving tracks, and dust, color may be obscured or distorted, resulting in a decrease in detection accuracy. Ying Y. et al. [4] proposed a real-time detection method for traffic cone targets based on the fusion of laser radar and vision. It uses a sensor algorithm integrated with laser radar and RGB cameras to detect the three-dimensional position and color information of traffic cones. The calculation complexity is low, but it is difficult to implement this system on automatic traffic cone retractors. Chen L. et al. [5] built a dual-camera experimental platform with a four-axis mechanical arm using cameras. The platform recognizes and grasps workpieces by positioning with dual-camera vision, but the cost of building a dual-camera vision positioning system with this camera is too high for mass production in automatic traffic cone retractors.

With the rise and rapid development of artificial intelligence, the performance of deep learning algorithms in target recognition has surpassed traditional image processing algorithms [6,7,8,9,10,11,12]. Object detection methods based on deep learning are divided into two-stage detection and single-stage detection. Two-stage detection is a process “from coarse to fine”, while single-stage detection is an “end-to-end” process [13]. An example of single-stage detection is the YOLOv5 network model proposed by Ultralytics in 2021, which has significant advantages in detection accuracy and inference speed and has been widely used in various target detection scenarios. Zhao Z. et al. [14] designed a YOLOv5 target detection algorithm suitable for cone color detection systems, achieving effective measurement of cone color recognition and distance. Lv H. et al. [15] proposed an improved YOLOv5 algorithm for traffic sign recognition with higher accuracy and better practical application. Han J. et al. [16] proposed a small target detection algorithm for UAVs (Unmanned Aerial Vehicles) based on YOLOv5, with a detection accuracy of 98.3% and a recall rate of 97.2%. CHEN Y. et al. [17] introduced a new attention mechanism module, Global-CBAM, into the YOLOv5 backbone, enhancing the network’s ability to detect small targets and improving its accuracy in traffic sign recognition applications. Although the detection accuracy of machine vision systems may be influenced by ambient weather and light, they are still widely used in the field of vehicles, roads, and traffic signs [18,19].

In this study, a YOLOv5 network model with high accuracy and a fast inference speed was deployed on an automatic traffic cone retractor to identify and locate traffic cones in real time, automatically adjusting the position and posture of the manipulator to collect cones, improving the automation level of the automatic traffic cone retractor, and realizing the application of machine vision in highway maintenance scenarios.

## 2. Mechanical Design of Automatic Traffic Cone Retractor

**Figure 1:**
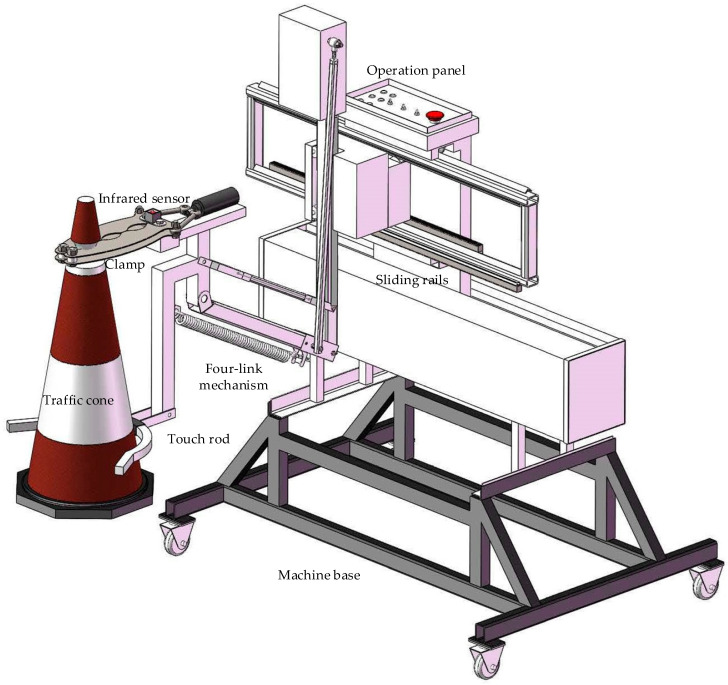
Schematic diagram of the automatic traffic cone retractor.

Figure 1 shows a schematic diagram of the structure of the compact integrated automatic traffic cone retractor. The mechanical structure mainly includes a manipulator, a clamp, a four-link mechanism, a gear rack slide rail, a touch rod, a base, and other parts.

The touch rod is used to capture and straighten the cones, and the base is equipped with universal wheels for multi-directional movement of the whole machine. The operation panel is equipped with multiple buttons for selecting operation modes and an emergency stop. The vehicle does not need any modification, as the whole machine is fixed to a carriage with bolts and can be folded up for storage in the vehicle when not in use, occupying a small volume.

The manipulator clamp is made up of four overlapping stainless-steel claws that are fastened with bolts. The rear part of the clamp is connected to the Direct Current (DC) electric push rod by the joint bolt, and the DC electric push-pull rod motor controls the extension and retraction of the piston rod to grip or release a cone with the clamp. The system automatically captures the cone signal and controls the manipulator clamp to grip a cone. The DC motor controls the four-link mechanism and the gear rack slide rail to move up, down, left, and right and transport a cone. A cone can be collected or released by driving the vehicle forward or backward. Through verification under actual working conditions, the stability and applicability of the automatic traffic cone retractor met the operational requirements [20].

## 3. Algorithm Improvements

### 3.1. Original YOLOv5 Algorithm

The YOLOv5 convolutional neural network is an object detection model that uses convolutional neural networks (CNNs) to perform and complete the tasks of object detection and classification.

**Figure 2:**
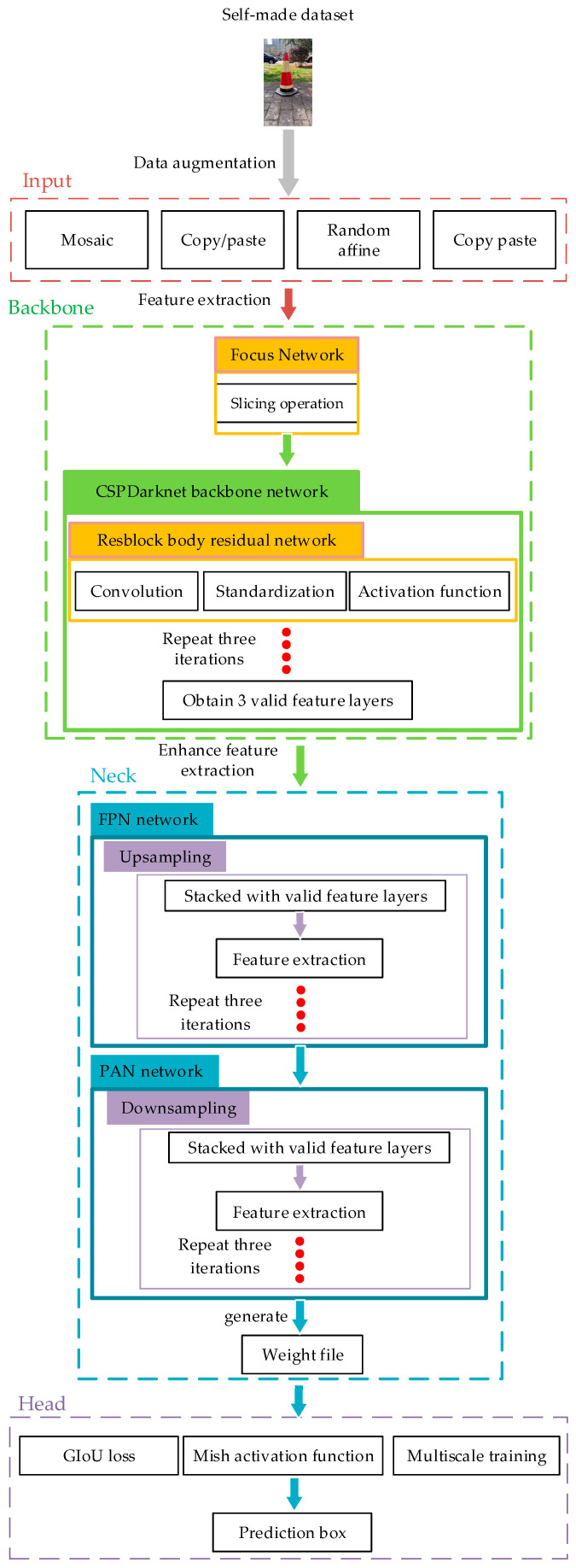
Structure of traffic cone perception system.

The process of using YOLOv5 for traffic cone recognition and position training and obtaining prediction boxes is shown in Figure 2.

The network structure consists of an input, a backbone, a neck, and a head. In the input, methods such as Mosaic, Copy/paste, Random affine, Mix-up, and Cutout are used to enhance the dataset, thereby improving the diversity, robustness, and generalization ability of the model.

The backbone added a focus network module, which expanded the input channels by a factor of four. The network performs slicing operations on input images, increasing the computational power of the network without losing information.

**Figure 3:**
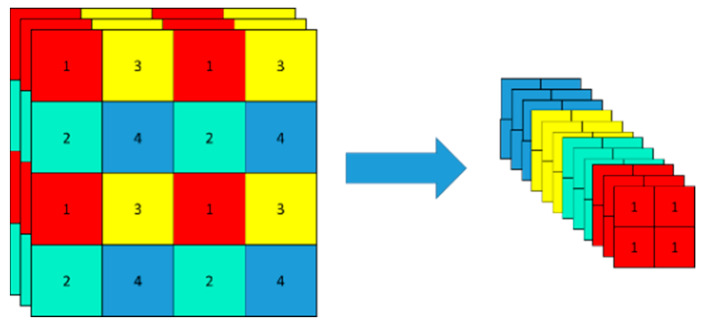
Slicing operation.

As shown in Figure 3, the focus module was replaced by a 6 × 6 convolutional layer. Although the computational complexity is equivalent, the convolutional layer is more efficient. The backbone uses CSPDarknet53 as its main network, which contains five Cross Stage Partial (CSP) Network modules. Based on the YOLOv5 backbone network Darknet53, this network was designed using the Cross Stage Partial Network structure to solve the problem of the computational complexity of inferences. It not only enhances the learning ability of the convolutional neural network, but it is also lightweight while maintaining accuracy, which reduces the network’s computational bottlenecks and memory costs.

The neck follows the FPN + PAN structure of the YOLO family, fusing feature maps of different levels through upsampling and downsampling operations to generate a multiscale Feature Pyramid Network (FPN). The FPN mainly achieves the fusion of features at different levels through upsampling and by fusing with coarser feature maps. The Path Aggregation Network (PAN) uses convolutional layers to fuse feature maps from different levels.

The head is used for target detection on the feature pyramid. This module adopts a method of multi-level feature fusion where the feature maps output by the backbone network are passed through a Conv module for channel dimension reduction and feature map scaling. Then, the feature maps of different levels are fused to obtain richer feature information, thereby improving detection performance. Additionally, the head incorporates GIoU loss, a Mish activation function, and multiscale training to further enhance detection accuracy.

### 3.2. Network Model for Traffic Cone Recognition and Positioning

When performing traffic cone retraction, the vehicle speed is generally controlled at 20 km/h, the cone retraction rate should reach 30 per minute, and the visual detection speed should be controlled at 6~10 fps/s to meet the above work speed. In addition, highway maintenance operations usually need to be carried out in various extreme weather conditions, at night, on curved roads, in dusty environments, and other scenarios, and external interference also has a significant impact on the recognition effect. To reduce the influence of external interference, the hardware adopts dust prevention and supplementary light to overcome the effect. When selecting a deep learning detection network, the real-time detection capability, accuracy, and anti-interference ability should all be guaranteed. In conclusion, during the operation of an automatic traffic cone retractor, real-time detection and positioning of a traffic cone’s position are required, with the system prioritizing frames per second (FPS) and average precision (AP) as key indicators.

YOLOv5-Lite is based on a series of ablative experiments using YOLOv5, which made its network structure lighter with faster operations and inferences, easier to deploy on embedded devices, and highly compatible with the operation requirements of automatic traffic cone retractors.

#### 3.2.1. Network Structure

**Figure 4:**
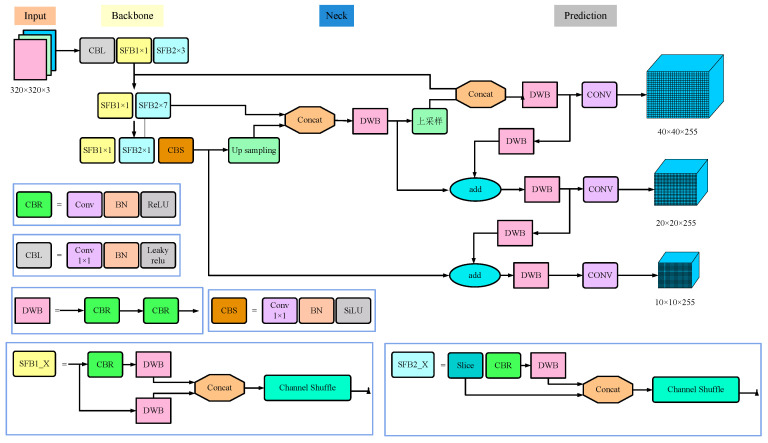
Network structure of YOLOv5-Lite.

YOLOv5-Lite removed the focus layer on the input side of the network based on YOLOv5 and eliminated four slicing operations, making it easier to deploy on embedded devices while maintaining acceptable model inference accuracy, as shown in Figure 4.

#### 3.2.2. Input

**Figure 5:**
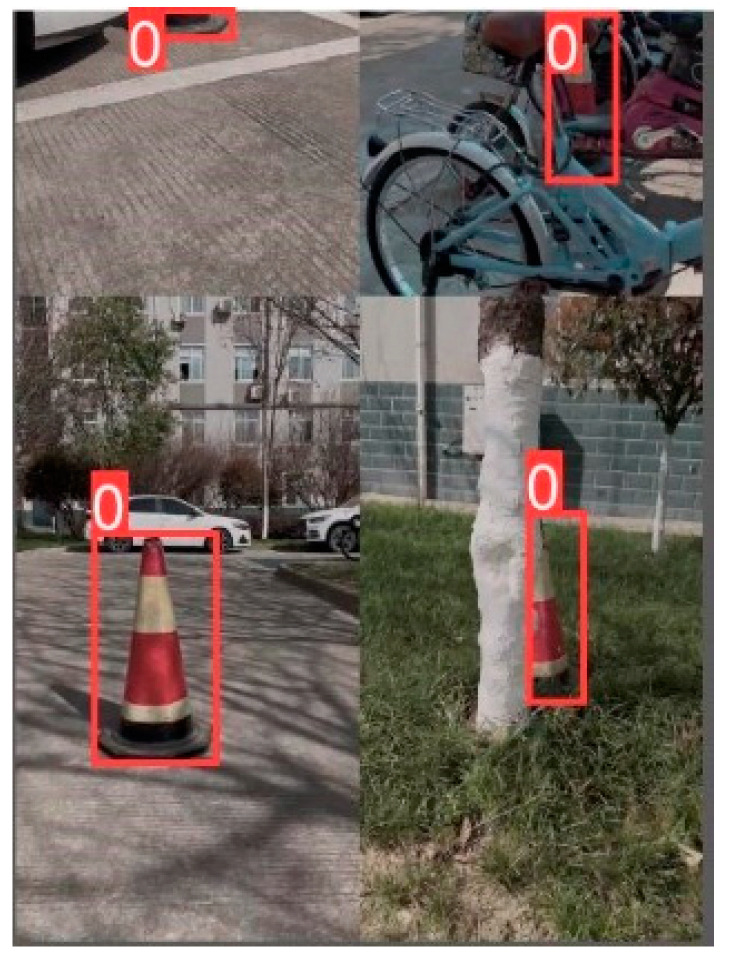
Data augmentation.

The input end of YOLOv5-Lite follows the Mosaic data augmentation of YOLOv5 and combines images via random zooming, cropping, and layout, as shown in Figure 5, which not only reduces GPU usage but also enriches datasets, providing a good strengthening effect for small object detection.

The photos in a dataset used for deep learning training must be rich and diverse, and the images must be of different sizes. Adaptive image scaling at the input end can effectively solve this problem. At the beginning of training, a uniform image size that meets the detection conditions is set. When a dataset is input into the network, it is scaled to the standard size, with black borders filling the blank areas, thereby optimizing the training effect while maintaining dataset diversity. The network also adopts adaptive computation of an anchor, selects the most suitable parameters using genetic algorithms, accelerates convergence, and ensures more adequate learning.

#### 3.2.3. Backbone

The focus layer network module in YOLOv5 compresses the network layers and gradients to reduce the number of calculations and improve network speed, but it has limitations and side effects. When the algorithm is deployed on embedded devices, using the focus module multiple times for slicing operations results in serious cache occupation and increased computational burden, greatly affecting the real-time performance of traffic cone recognition and positioning. Therefore, YOLOv5-Lite replaces the focus module with a convolutional layer to speed up the network while also achieving better performance.

Most of the convolutional neural networks for image classification have achieved good recognition results, such as LeNet, VGGNet, AlexNet, etc., but they also face some common problems, including large models, numerous parameters, and high computational complexity, making them difficult to apply on mobile or embedded devices. Due to the above reasons, shuffle Net was selected as the backbone network for the system [21]. This network combines group convolution and pointwise convolution in pointwise group convolution, which not only balances detection accuracy and speed well but also significantly reduces the computational complexity of the network. However, after pointwise group convolution, the feature maps obtained by different groups are only related to the corresponding group inputs, which easily leads to very limited learning features and information loss. Therefore, shuffle Net further introduces a channel shuffle mechanism, which extracts channels from the feature maps of different groups and combines them to solve the above problem.

The backbone also uses a C3 layer, which is an important module in the YOLOv5 network architecture, playing a crucial role in improving model performance and computational efficiency. The C3 layer is simpler, faster, and lighter compared to the BottleneckCSP in the original version and can achieve better results with similar losses. The C3 layer uses multi-branch separable convolution. If the C3 layer is used frequently or with a high number of channels, the system will occupy more cache space and reduce the running speed. Therefore, the backbone of YOLOv5-Lite avoids using the C3 layer multiple times and does not use a high-channel C3 layer.

#### 3.2.4. Neck

While YOLOv5-Lite continues to use the FPN + PAN structure of YOLOv5′s neck part, the head part of YOLOv5 was pruned based on four efficient network design principles [22] and uses the same number of channels to optimize memory access and usage. YOLOv5-Lite is divided into four models with different depths and widths of feature extraction: YOLOv5-Lite-e, YOLOv5-Lite-s, YOLOv5-Lite-c, and YOLOv5-Lite-g. To ensure that the FPS remained stable at around 10 after deployment on embedded devices, the pre-trained weights of the smaller models (YOLOv5-Lite-e and YOLOv5-Lite-s) were selected.

## 4. Experiments and Analysis of Results

### 4.1. Dataset Creation and Training Environment

**Figure 6:**
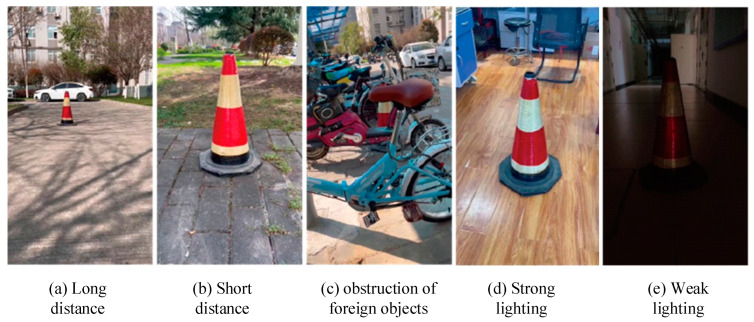
Images of cones obtained under different conditions.

During highway maintenance operations, the distance between traffic cones is generally 8–10 m. When capturing images for the dataset, images between 1 and 5 m were classified as short distances, and images between 5 and 10 m were classified as long distances. Additionally, considering sudden situations such as highway traffic accidents and temporary repairs, as well as various types of extreme weather, dust, and nighttime work conditions, photos were also taken with strong and weak lighting and with cones obstructed by foreign objects. The images of these scenarios were compiled into a photo set, as shown in Figure 6.

**Figure 7:**
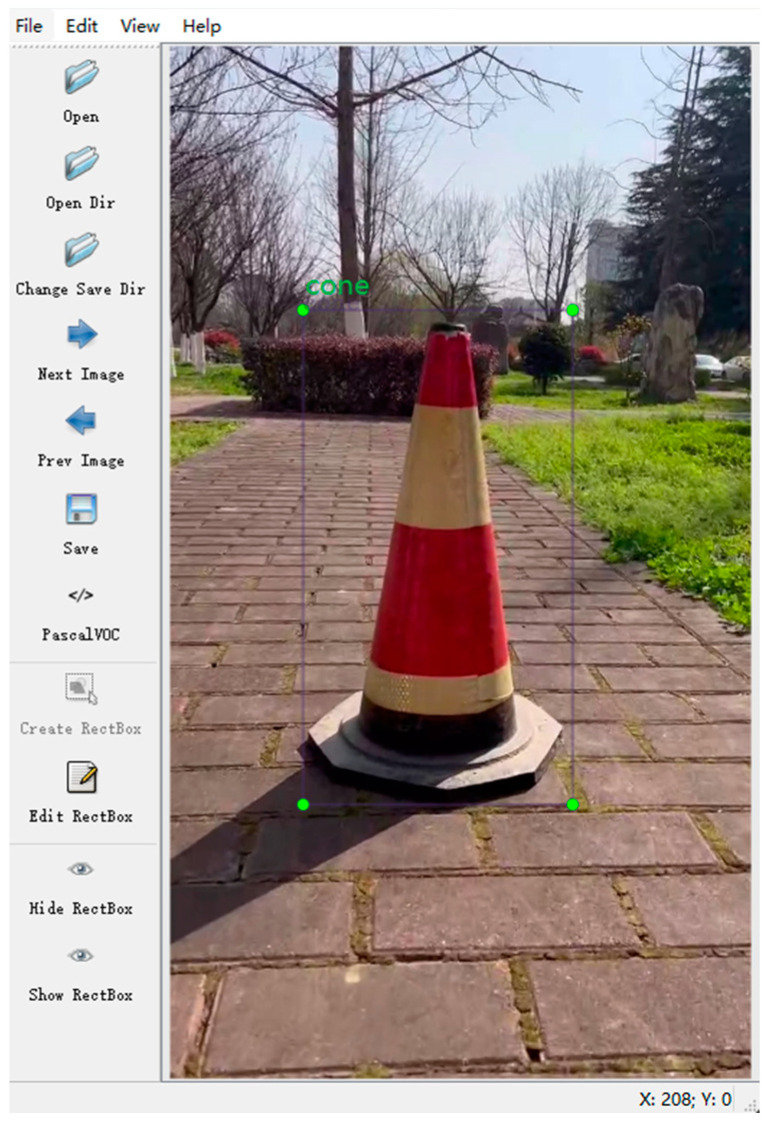
Target annotation with Labelimg.

The system used the open-source tool Labelimg to label the photo set, as shown in Figure 7, labeling the traffic cones as “cone”, generating a corresponding. xml tag file, and creating a COCO dataset in VOC label format. The completed dataset consisted of 1080 items, with each of the five scenarios accounting for one-fifth of the total. Subsequently, PyTorch was used to build a deep learning framework. The computer configuration used for deep learning network training included an Intel(R) Core(TM) i9-10900X CPU @ 2.30 GHz, an NVIDIA GeForce PTX3080, a 10 GB graphics processing unit (GPU), 64 GB of memory, and a CUDA 11.3 acceleration environment.

### 4.2. Analysis of Results

#### 4.2.1. Network Performance Evaluation Indicators

When the automatic traffic cone retractor was operating, the speed of cone placement and retraction was required to reach 30 cones per minute. Therefore, the recognition effect of a model trained using different networks was evaluated using precision, recall, frames per second (FPS), and model size [23,24,25,26,27]. The specific formulas of the indexes are as follows:

(1) $$Precision=\frac{TP}{TP+FP}$$

(2) $$Recall=\frac{TP}{TP+FN}$$

(3) $$mAP=\frac{1}{n}\sum_{i=1}^{n}\int_{0}^{1}p\left(R\right)dR$$

In Equations (1)–(3), *TP* is true positive, which means an actual positive sample is predicted to be a positive sample; *FP* is false positive, which means an actual negative sample is predicted to be a positive sample; and *FN* is false negative, which means an actual positive sample is predicted to be a negative sample. The higher the precision and recall, the higher the detection accuracy of the model. *mAP* is the mean average precision of each category. First, the precision and recall are calculated at different confidence thresholds and a P-R curve is generated. Then, the area surrounded by the curves of each category is calculated and divided by the number of categories to obtain the *mAP* value. *mAP*_0.5 refers to the average precision when the IOU threshold is 0.5. When the automatic traffic cone retractor is operating, it pays great attention to the speed and flexibility of the network model, so *mAP*_0.5 is also an important evaluation indicator.

#### 4.2.2. Network Model Training and Comparison

**Figure 8:**
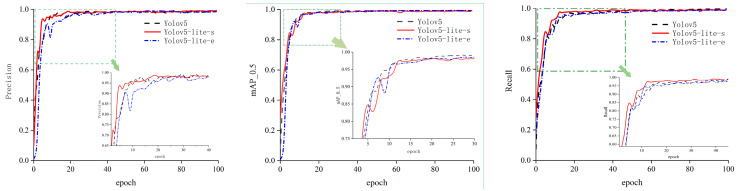
Changes in precision, recall, and *mAP*_0.5.

YOLOv5 used YOLOv5-s pre-trained weights, while YOLOv5-Lite used YOLOv5-Lite-s and YOLOv5-Lite-e pre-trained weights for training, and the training epoch was set to one hundred. The changes in the evaluation metrics precision, recall, and *mAP*_0.5 during the training processes of the different network models are shown in Figure 8. It can be seen in the figure that the recognition accuracy of the three types of networks could reach around 95%, with YOLOv5 having the most stable accuracy and YOLOv5-Lite-e having larger fluctuations. YOLOv5-Lite-s had the fastest convergence speed for recall, followed by YOLOv5.

The evaluation indicator *mAP*_0.5 shows that YOLOv5 was the best network model among the three at balancing speed and accuracy, and its convergence speed was also the fastest. YOLOv5-Lite-s was slightly inferior but could also meet the requirements of single-target traffic cone recognition.

**Figure 9:**
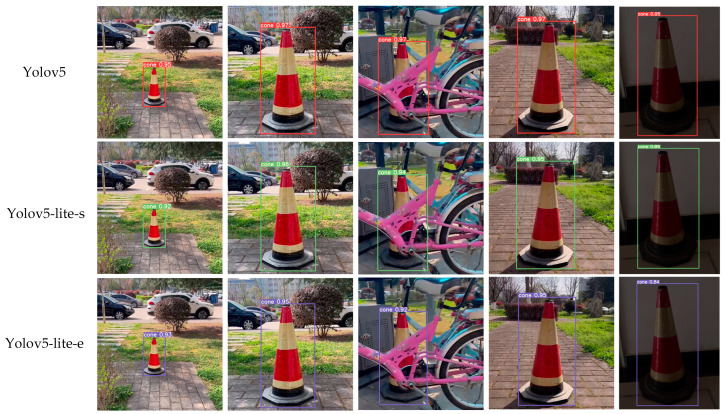
Recognition effects of three networks under five different working conditions.

The trained network models were used to identify and locate the traffic cones in the dataset, which simulated five different working conditions, including long distances, short distances, occlusion, strong lighting, and weak lighting. The test dataset and the training dataset are the same set of data. The results are shown in Figure 9, with confidence levels of 95%, 97%, 97%, 97%, and 95% for YOLOv5; of 92%, 96%, 94%, 95%, and 89% for YOLOv5-Lite-s; and of 93%, 95%, 92%, 95%, and 84% for YOLOv5-Lite-e. From the detection results, it can be seen that YOLOv5 could maintain an accuracy of over 95% under each working condition. YOLOv5-Lite-s could maintain an accuracy of around 90%, while the accuracy of YOLOv5-Lite-e fluctuated greatly, even falling below 85% in weak lighting conditions, making it difficult to meet the requirements for stable recognition and positioning of an automatic traffic cone retractor.

According to the detection results of the network performance evaluation indicators precision, recall, and *mAP*_0.5 during the training process, it can be seen that the YOLOv5 and YOLOv5-Lite-s networks could meet the requirements of recognition accuracy of an automatic traffic cone retractor under different working conditions.

#### 4.2.3. Model Deployment and Comparison

**Figure 10:**
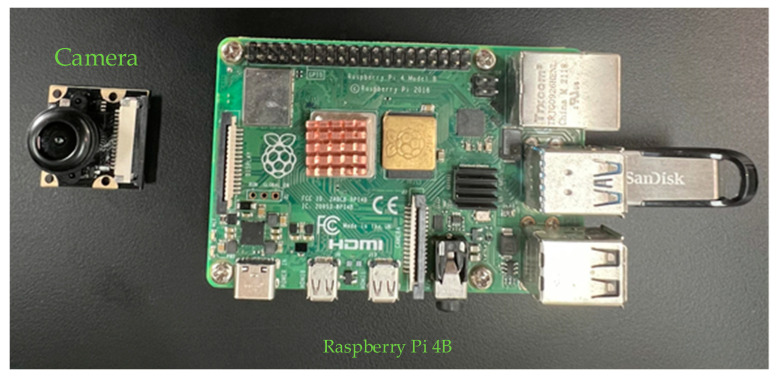
Raspberry Pi 4B development board.

The number of frames transmitted per second during real-time detection by a network model is a key indicator of the selected model. As shown in Figure 10, the embedded device used a Raspberry Pi 4B development board ( Manufactured by Raspberry Pi Foundation, bought from AN AVNET COMPANY, Shanghai, China),the configured 1.5 GHz quad-core CPU of which could run lightweight neural network models. The RS232 communication port could interact with the PLC control system of the automatic traffic cone retractor.

**Figure 11:**
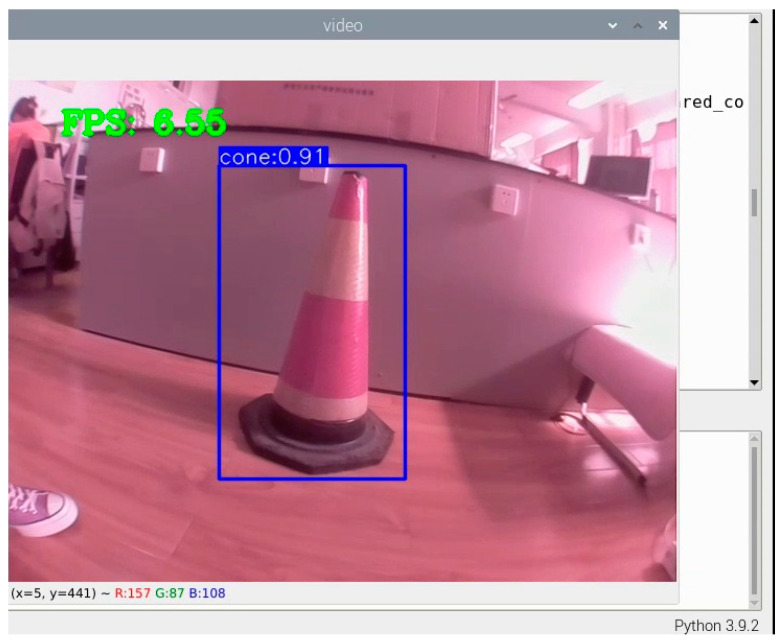
Real-time detection effect.

The best weight file obtained from the model training was converted to an onnx file and transferred to the Raspberry Pi 4B. The compiled detection program used the officially adapted camera for a real-time detection effect, as shown in Figure 11.

The three models (YOLOv5-s, YOLOv5-Lite-s, and YOLOv5-Lite-e) were deployed on the Raspberry Pi 4B development board for identification experiments with traffic cones in different application scenarios. First, the cone recognition video was recorded, and each video was intercepted for 10 s. Then, the intercepted videos were intercepted again at intervals, 100 frames were extracted from each video, and the confidence and FPS of each video frame were recorded. Finally, the average frame rate and confidence level of each network were calculated based on the recorded data. The data are shown in Table 1, where the size of the weight file represents the size of the corresponding network model. The average accuracy of the YOLOv5-s network reached 92.5%, but the detection speed struggled to meet the real-time requirements of the automatic traffic cone retractor. The detection accuracy of the YOLOv5-Lite-e network was only 83.5% and decreased under low-light conditions, making it struggle to handle operations in extreme weather. With an average detection accuracy close to 90%, YOLOv5-Lite-s could achieve a detection speed of 9.22 fps. This algorithm could maintain high recognition accuracy in the five simulated operation scenarios and was well-suited for the operational requirements of the automatic traffic cone retractor.

#### 4.2.4. Experimental Results and Analysis of Experimental Platform Application

**Figure 12:**
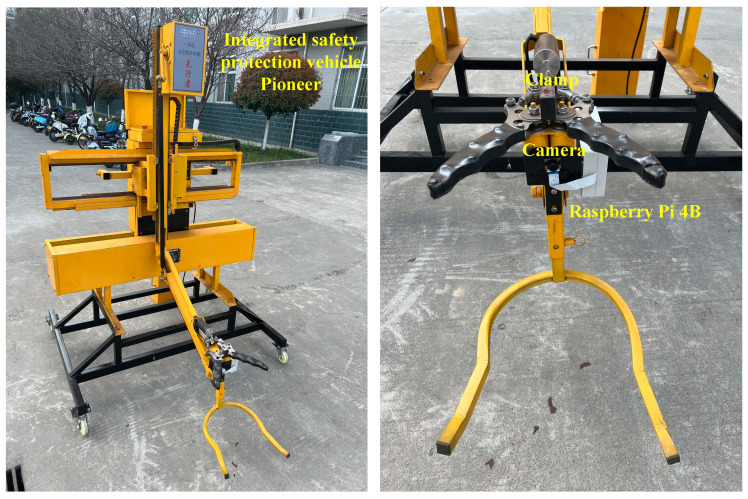
The experimental platform of automatic traffic cone retractor.As shown in Figure 13, when the operation mode is traffic cone retraction, the four-link mechanism’s motor lowers the manipulator to the retraction height. Once the cone enters the clamping range, the ultrasonic sensor in front of the manipulator feeds an electrical signal back to the PLC, and the machine vision equipment starts tracking and detecting. It transmits the traffic cone coordinates and contour information to the PLC. Subsequently, the PLC drives the four-link mechanism, gear rack slide rail, and electronic push-pull rod to move, adjusting the width of the manipulator’s extension and the vertical position, as well as the angle of the clamp. Finally, the clamp grips the cone, and the manipulator lifts the cone to the upper limit. After the infrared sensor above the clamp detects a human hand, the clamp releases the cone. The operator can then remove the cone, and the manipulator moves to the lower limit for the next recovery operation.

The experimental platform was manufactured according to its design scheme, as shown in Figure 12. The center of the Raspberry Pi camera and the manipulator clamp were at the same point.

When the operation mode is cone deployment, with the manipulator in the lower limit position, the operator places a traffic cone in the clamp, and the manipulator transports the cone downward after the fixture clamps the cone. When the manipulator moves to the lower limit, the clamp is released, and the traffic cone is placed smoothly on the ground.

Experiments were conducted with different colors and types of traffic cones placed at equal intervals in the test area. The experiments were carried out under different conditions such as normal scenes, low lighting, cone obstruction, low lighting with obstruction, continuous operation, etc., to verify the accuracy, continuity, and anti-interference ability of the experimental platform in traffic cone retraction operations. If a traffic cone was not located or picked up successfully, it was counted as a failure. The number of failures in 30 sets of experiments was recorded. In normal scenes, only one cone was not recognized. In low-light scenes, there was one failure due to a recognition error. When the traffic cones were obstructed, there were two failures under normal lighting and three failures in weak lighting. The rest of the time, the equipment worked well and the retrieval operation proceeded smoothly.

The test results show that the experimental platform of the automatic traffic cone retractor operated stably, the traffic cone recognition and positioning system ran smoothly, and the control system could accurately complete the traffic cone retrieval action and meet the requirements of highway maintenance operations.

## 5. Discussion

This article compared and analyzed the advantages and disadvantages of three different network models (YOLOv5-s, YOLOv5-Lite-s, and YOLOv5-Lite-e) that were applied to the operation of an automatic traffic cone retractor. Based on the recognition results of the models, it can be concluded that the YOLOv5-s network model had the highest recognition accuracy and recall rate. Its accuracy was above 90% under five different working conditions, and the model could effectively handle various extreme situations during cone retraction. The YOLOv5-Lite-e network model had large fluctuations in recognition accuracy when encountering extreme weather conditions such as weak lighting, making it struggle to meet the basic requirements of stable recognition during operation. Although the recognition accuracy and recall rate of the YOLOv5-Lite-s network model were not as high as those of YOLOv5-s, the recognition stability in various working conditions could meet the operational requirements. In addition, the average frame rate of the YOLOv5-s network model was only 5.75 fps.

It is difficult to guarantee that this model can perform the recognition and positioning tasks in real time. However, YOLOv5-Lite-s’s average confidence and frame rate values can meet the speed and accuracy requirements of the automatic traffic cone deployment machine for cone recognition and positioning.

Finally, after experimental verification, the algorithm of the deep learning neural network YOLOv5-Lite-s was integrated into the automatic traffic cone retractor, the control system and the traffic cone recognition and positioning system worked stably and accurately, and occasionally missed cones could be corrected through simple manual cooperation. The traffic cone retractor not only ensures the safety of drivers but also effectively improves the automation level of traffic cone placement and retraction, filling a gap in the application of machine vision to automatic traffic cone retraction.

In future research, we will adopt higher versions such as YOLOv8 or YOLOv10 to further improve the recognition accuracy and real-time performance of traffic cones. In addition, we will develop a type of intelligent traffic cone with remote control and wireless charging functions, which can realize the linkage operation of the five traffic cones to the designated position and complete the straight or diagonal road closure.

## Figures and Tables

**Figure 13:**
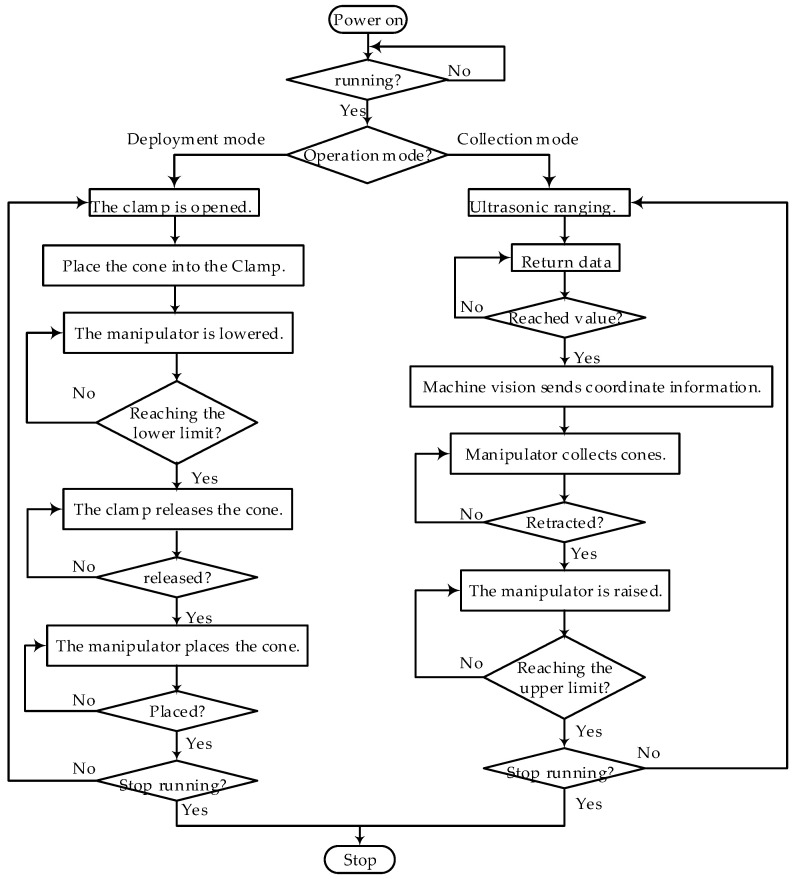
Workflow diagram of the automatic traffic cone retractor.

**Table 1:** Real-time detection data of various networks.

Category	Model Size	Average Frame Rate Title 3	Average Confidence
YOLOv5-s	33.6 m	5.75	0.925
YOLOv5-Lite-s	5.88 m	9.22	0.896
YOLOv5-Lite-e	2.77 m	10.54	0.835

## Data Availability

The data presented in this study are available on request from the corresponding author.
